# Mindfulness-Based App to Reduce Stress in Caregivers of Persons With Alzheimer Disease and Related Dementias: Protocol for a Single-Blind Feasibility Proof-of-Concept Randomized Controlled Trial

**DOI:** 10.2196/50108

**Published:** 2023-10-13

**Authors:** Emily C Woodworth, Ellie A Briskin, Evan Plys, Eric Macklin, Raquel G Tatar, Jennifer Huberty, Ana-Maria Vranceanu

**Affiliations:** 1 Center for Health Outcomes and Interdisciplinary Research Massachusetts General Hospital Boston, MA United States; 2 Harvard Medical School Boston, MA United States; 3 Biostatistics Center Massachusetts General Hospital Boston, MA United States; 4 Healthy Minds Innovations Madison, WI United States; 5 Department of Medicine University of Texas Health San Antonio, TX United States

**Keywords:** caregiver, dementia, mindfulness, mobile app, protocol, randomized controlled trial

## Abstract

**Background:**

Informal caregivers (ie, individuals who provide assistance to a known person with health or functional needs, often unpaid) experience high levels of stress. Caregiver stress is associated with negative outcomes for both caregivers and care recipients. Mindfulness-based interventions (MBIs) show promise for improving stress, emotional distress, and sleep disturbance in caregivers of persons with Alzheimer disease and related dementias (ADRD). Commercially available mobile mindfulness apps can deliver MBIs to caregivers of persons with ADRD in a feasible and cost-effective manner.

**Objective:**

We are conducting a single-blind feasibility proof-of-concept randomized controlled trial (RCT; National Institutes of Health [NIH] stage 1B) comparing 2 free mobile apps: the active intervention Healthy Minds Program (HMP) with within-app text tailored for addressing stress among caregivers of persons with ADRD, versus Wellness App (WA), a time- and dose-matched educational control also tailored for caregivers of persons with ADRD.

**Methods:**

We aim to recruit 80 geographically diverse and stressed caregivers of persons with ADRD. Interested caregivers use a link or QR code on a recruitment flyer to complete a web-based eligibility screener. Research assistants conduct enrollment phone calls, during which participants provide informed consent digitally. After participants complete baseline surveys, we randomize them to the mindfulness-based intervention (HMP) or educational control podcast app (WA) and instruct them to listen to prescribed content for 10 minutes per day (70 minutes per week) for 12 weeks. Caregivers are blinded to intervention versus control. The study team checks adherence weekly and contacts participants to promote adherence as needed. Participants complete web-based self-report measures at baseline, posttest, and follow-up; weekly process measures are also completed. Primary outcomes are a priori set feasibility benchmarks. Secondary outcomes are stress, emotional distress, sleep disturbance, caregiver burden, mindfulness, awareness, connection, insight, and purpose. We will calculate 1-sided 95% CI to assess feasibility benchmarks. Effect sizes of change in outcomes will be used to examine the proof of concept.

**Results:**

Recruitment started on February 20, 2023. We have enrolled 27 caregivers (HMP: n=14; WA: n=13) as of June 2023. Funding began in August 2022, and we plan to finish enrollment by December 2023. Data analysis is expected to begin in May 2024 when all follow-ups are complete; publication of findings will follow.

**Conclusions:**

Through this trial, we aim to establish feasibility benchmarks for HMP and WA, as well as establish a proof of concept that HMP improves stress (primary quantitative outcome), emotional distress, sleep, and mindfulness more than WA. Results will inform a future efficacy trial (NIH stage II). HMP has the potential to be a cost-effective solution to reduce stress in caregivers of persons with ADRD, benefiting caregiver health and quality of care as well as patient care.

**Trial Registration:**

ClinicalTrials.gov NCT05732038; https://clinicaltrials.gov/study/NCT05732038

**International Registered Report Identifier (IRRID):**

DERR1-10.2196/50108

## Introduction

### Background

More than 1 in 5 Americans (ie, approximately 53 million) are informal caregivers [1]. These individuals provide assistance to known persons with physical, mental, or cognitive illness or disability, often unpaid [2]. Almost half of caregivers who provide help to older adults care for someone with Alzheimer disease or related dementias (ADRD) [3]. Caregivers have been dubbed “the hidden patients” because the negative physical and mental health consequences of caregiving are often underrecognized and undertreated [4]. Nearly 60% of caregivers of persons with ADRD experience high levels of psychological stress, and more than 1 in 3 caregivers of persons with ADRD report that their health has deteriorated due to caregiving responsibilities [5]. Some key sources of caregiver stress include ongoing worry about their loved one’s health, concerns about whether they are doing the caregiving job well, challenges navigating regular life in addition to caregiving responsibilities, concerns about the impact of caregiving on their own health, social isolation, and financial strain [6,7]. High caregiver stress is associated with mental health concerns [8], sleep difficulties [9], and an increased risk for morbidity and mortality [10]. In previous studies, 42%-55% of caregivers reported looking for feasible and effective stress management interventions [11]; yet this population often has limited time for outpatient psychotherapy. Given the negative consequences of caregiver stress on the health and well-being of both caregivers and care recipients, decreasing caregiver stress in an accessible delivery format is a public health concern.

Mindfulness-based interventions (MBIs) show promise as an efficacious nonpharmacological treatment to reduce caregiver stress [2]. Mindfulness meditation is defined as paying attention on purpose, in the present moment, nonjudgmentally [12,13]. Practicing mindfulness is thought to help individuals cultivate acceptance and inner peace, regardless of challenging life circumstances [14-16]. Across many populations, the practice of mindfulness meditation is associated with decreased stress [17,18], improved emotional functioning [19,20], and improved sleep [21,22]. Several recent systematic reviews have supported the effectiveness of MBIs for improving stress, emotional functioning, and sleep in caregivers, including caregivers of persons with ADRD [2,14,17,18,21,23-27].

Although research has established the benefits of MBIs for caregivers, the prevailing models of delivery of these interventions fall short for several reasons. First, many of these programs are conducted in person [28], which is sometimes not feasible for caregivers’ schedules (eg, needing respite for care recipients) [7]. Second, even when programs have been delivered remotely, these often follow a synchronous format (ie, caregivers must attend remotely at the same time each week), which is challenging given the unpredictability of caregiving responsibilities. Third, traditional programs typically demand substantial amounts of continuous time that caregivers are unable to commit (eg, a traditional mindfulness-based stress reduction program involves 8 weekly 2.5- to 3.5-hour-long sessions as well as 45 minutes of prescribed practice per day) [29]. Fourth, traditional programs require trained providers who are often costly, are not usually covered by insurance, and are not always readily available [30-32]. More feasible, scalable, and cost-effective solutions are needed to deliver MBIs to caregivers.

Mobile mindfulness apps may be uniquely positioned to address the aforementioned barriers by allowing caregivers to practice meditation on their own schedule at little or no cost. The majority of caregivers own a smartphone and regularly use mobile apps [33]. Additionally, adults aged 50 years or older, who represent a sizable portion of caregivers of persons with ADRD, are the fastest-rising users of smartphone technology in the past decade [34]. In qualitative studies, caregivers express interest in using mobile apps for stress management and health information [35] including to learn mindfulness meditation for stress management [36-39]. To the best of our knowledge, there are no consumer-based mobile apps with proven efficacy in decreasing stress, emotional distress, and sleep difficulties among caregivers of persons with ADRD [40]. Although mindfulness-based mobile apps show promise for helping caregivers of persons with ADRD decrease stress, emotional distress, and sleep difficulties, additional research is needed to establish feasibility and efficacy before widespread dissemination.

Healthy Minds Program (HMP) is a mindfulness-based mobile app that may be well suited for use with caregivers of persons with ADRD for several reasons. First, HMP is unique because it teaches mindfulness skills grounded in theory [41] with empirical support [42-44]. Having a theoretical underpinning is important because it allows the app to be a vehicle for the delivery of evidence-based mindfulness skills. Second, previous research has shown that HMP reduced emotional distress and burnout in firefighters [44]; improved psychological distress, stress, social connectedness, and mindfulness in adults [43]; and reduced stress and increased mindfulness, self-compassion, and human flourishing in medical students [42]. Third, HMP is free of charge, which increases its reach especially among caregivers who often experience financial burdens because of their role. Fourth, HMP has customizable features (eg, a diverse set of voices to choose from) that have the potential to increase acceptability across race, ethnicity, and gender. Finally, HMP supports content tailoring to include on-screen language and scenarios that are relevant for caregivers of persons with ADRD.

Here, we will be conducting a National Institutes of Health (NIH) stage 1B trial (NCT05732038) comparing HMP with a time- and dose-matched educational control app, Wellness App (WA). Our team tailored the content of both apps to highlight case scenarios specific to caregivers of persons with ADRD. Both interventions (HMP and WA) consist of 12 weeks of guided listening for 10 minutes per day. Participants complete outcome measures at baseline, posttest, and follow-up (2 months after posttest), as well as weekly process measures.

### Objectives

The purpose of this paper is to describe the protocol and status of our ongoing single-blind feasibility proof-of-concept randomized controlled trial (RCT) of HMP versus WA in caregivers of persons with ADRD. Our primary hypothesis is that HMP and WA will meet a priori feasibility markers (ie, acceptance and demand benchmarks). Our secondary hypothesis is that participation in HMP will be associated with a greater decrease in caregiver stress (primary quantitative outcome), emotional distress, sleep dysfunction, and caregiver burden, and a greater increase in mindfulness, awareness, connection, insight, and purpose (secondary quantitative outcomes) compared with WA, and improvement will be sustained through the 20-week follow-up. Results will directly inform an NIH stage 2 fully powered RCT of HMP versus WA.

## Methods

### Study Design and Setting

**Figure 1:**
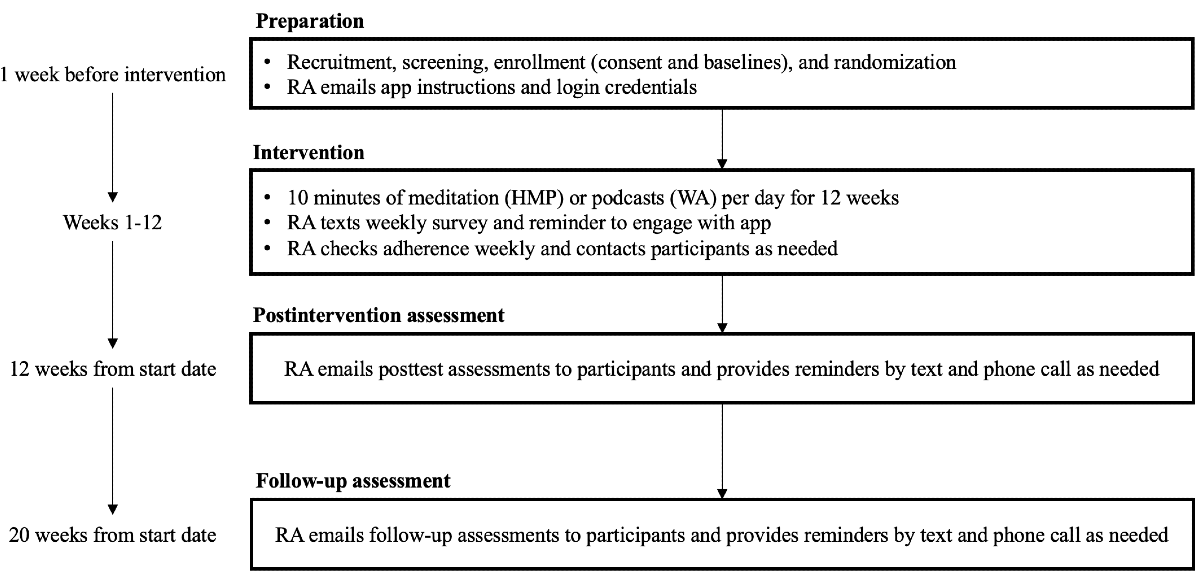
Study design and timeline. HMP: Healthy Minds Program; RA: research assistant; WA: Wellness App.

This NIH stage 1B, single-blind, feasibility, proof-of-concept RCT is testing HMP versus WA (time- and dose-matched control) in caregivers of persons with ADRD. Data collection is underway (started in February 2023; n=27 caregivers as of June 2023). We plan to recruit 80 caregivers of persons with ADRD reporting elevated levels of stress. Our institutional review board approved this study (2022P001601). Figure 1 provides an overview of the study design and timeline.

### Inclusion and Exclusion Criteria

Table 1 presents the study inclusion and exclusion criteria and rationale. These eligibility criteria were informed by previous MBI studies with caregivers, including those of persons with ADRD [2,14,17,18,21,23-27], as well as a previous study with caregivers funded by the NIH (NCT00177489). The criteria are meant to be as inclusive as possible to maximize generalizability by allowing individuals who self-identify as caregivers to participate rather than requiring a certain number of hours spent caregiving, specific responsibilities, or other criteria.

**Table 1:** Study inclusion and exclusion criteria and rationale.

Criteria			Rationale
**Inclusion**			
	Age 18 years or older	Most caregivers are adults; study procedures are formulated for adults only	
	English fluency and literacy	Measures, instructions, and app are in English	
	Meeting criteria for being a caregiver (ie, family or friend of a care recipient who provides care not through an agency) [2]	Population	
	Perceived Stress Scale (4 items) version ≥6 [45]	Population; ensure that the intervention is needed (not all caregivers experience caregiver stress)	
	Willing to be randomized	Necessary for scientific rigor	
	Care recipient must be rated >1 on the Functional Assessment Staging Tool [46]	Population; ensure that caregivers have a threshold of caregiving tasks	
**Exclusion**			
	Any planned change in psychotropic pharmacologic treatment for the duration of the study	Treatment confound	
	Use of any consumer-based mindfulness meditation app for more than 60 minutes per month in the past 6 months	Treatment confound	
	Current participation in a meditation program (eg, mindfulness-based stress reduction, mindfulness-based cognitive therapy, etc)	Treatment confound	
	Major illness anticipated to worsen dramatically or require surgery in the next 20 weeks (study duration)	Treatment confound	
	Active treatment for cancer (chemotherapy or radiation)	Treatment confound	
	Imminent placement of care recipient in a nursing home or with another caregiver (within 4 months)	Treatment confound	
	Involvement in another clinical trial for caregivers	Treatment confound	
	For participants aged 65 years and older: Short Portable Mental Status Questionnaire: ≥4 errors [47]	Validity and feasibility	

### Recruitment and Screening

For this trial, we are focused on recruiting geographically, racially, and ethnically diverse informal caregivers to ensure generalizability. We have established partnerships with a variety of organizations dedicated to supporting caregivers and care recipients (eg, National Alliance of Caregiving and Boston Ministerial Alliance) that share our recruitment materials (eg, flyer) on social media and listservs. Additionally, we directly contact caregivers from our previous nonclinical trial studies that indicated interest in learning about additional research opportunities. Interested caregivers use a link or QR code on flyers, social media ads, and email blasts to fill out a web-based eligibility questionnaire through REDCap (Research Electronic Data Capture; Vanderbilt University) [48]. Caregivers who want more information on the study can contact the research assistant (RA) before completing the screening in REDCap. Ineligible caregivers receive an on-screen message at the end of the screening in REDCap notifying them of their ineligibility along with a link to a resource sheet. For participants who meet initial eligibility criteria, a final survey page appears where they confirm their interest in participating and provide suitable times for a 20- to 30-minute enrollment phone call. During this call, the RA administers the Short Portable Mental Status Questionnaire (SPMSQ; for those over the age of 65 years) [47] and the Functional Assessment Staging Tool (FAST) [46] to finalize eligibility criteria.

### Ensuring Validity of Participant Data by Avoiding “Imposter” Participants

Previous research has shown that remote studies have an increased risk of enrolling “imposter” participants (ie, participants who misrepresent themselves to appear eligible for a study) [49,50]. In a web-based study using MTurk (a crowdsourcing service), Hydock [49] found that more than 12% of participants falsified their identities “for the chance of financial gain.” To address this concern, our team has identified “red flags” that suggest that a participant is misrepresenting their identity and requires additional attention and screening, including (1) large volumes of back-to-back inquiries following a similar format (eg, all emails are firstnamelastname ####@gmail.com), (2) spelling mistakes and grammatical errors, (3) incorrect or inconsistent survey responses (eg, one participant wrote they heard about this study on Craigslist), (4) providing out-of-service or Google Voice (Google LLC) numbers (the use of Google Voice numbers has been cited as a commonality among many imposter participants [51]), (5) poor sound quality during phone calls, and (6) exaggerated or illogical statements during phone calls (eg, stating, “I take my father with advanced dementia to the gym and we work out together”). These are not exclusion criteria but rather indicate to the research team that it is possible that the inquiry about the study was made by a potential imposter participant.

Our team has developed a protocol to prevent imposter participants from enrolling. First, when participants inquire about the study on public-facing platforms (eg, “Rally,” a web-based platform for advertising research studies across our hospital system), we call them before sending the link to the screening survey. This has been cited in other studies as a strategy to eliminate imposter participants [51]. Second, we have used a “three-strike” system. Once a potential participant has displayed 3 red flags (described above), they are no longer considered eligible for this study. We move these participants to the “suspected imposter” section of our log and do not continue contact attempts; we have noticed that the more we engage with potential imposter participants, the more false inquiries we receive. Third, we do not provide compensation for the completion of baseline assessments; instead, our compensation is divided up between posttest and follow-up, which discourages imposter participants who might enroll for the baseline compensation and then drop out. So far, we have identified 44 suspected imposter participants (approximately 36%) out of the 123 inquiries we have received. We acknowledge that these procedures have the potential to exclude eligible participants (ie, false negatives); however, as a team, we agreed to prioritize the integrity of the data for this small-scale feasibility RCT.

### Enrollment

**Figure 2:**
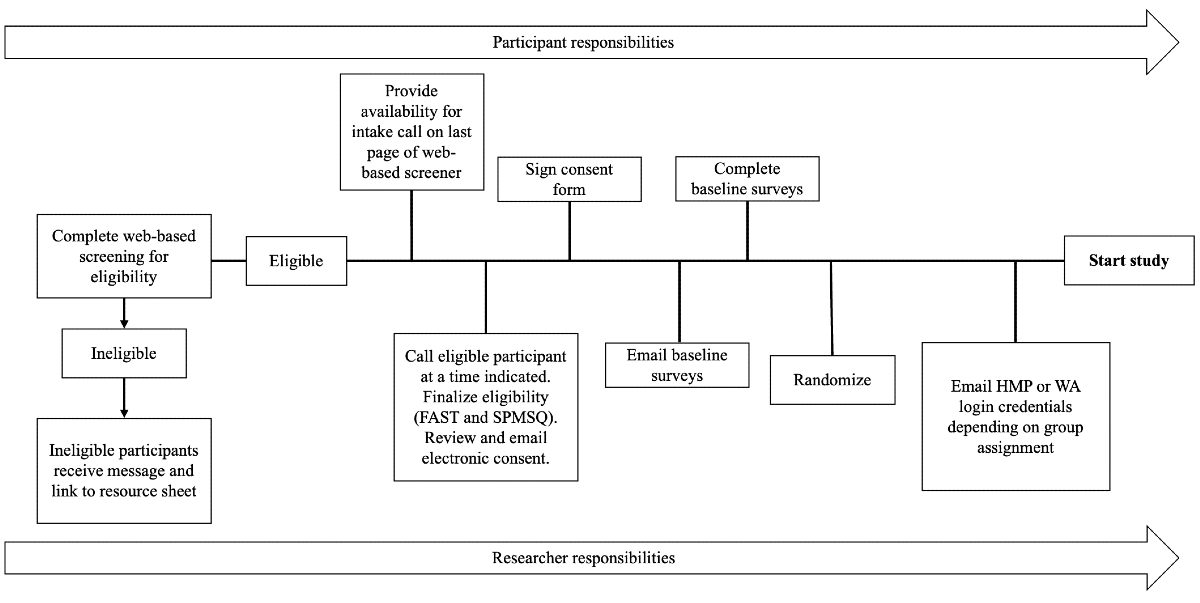
Recruitment and enrollment procedures. FAST: Functional Assessment Staging Tool; HMP: Healthy Minds Program; SPMSQ: Short Portable Mental Status Questionnaire; WA: Wellness App.

If participants are deemed eligible during the enrollment call, the RA reviews the consent form, answers any questions, and assists the participant with signing the consent form by electronic consent on REDCap. Once the consent form has been signed, the RA emails baseline surveys to the participant through REDCap. Study staff randomize the participants after baseline completion. Our senior biostatistician programmed randomization into REDCap and stratified by gender (given that caregivers tend to be women) [1]. The RA emails the participant instructions to download and login to their assigned app (HMP or WA). To protect participant confidentiality, the study team creates accounts with study-specific login email addresses (eg, mghstridestudy+1@gmail.com and mghstridestudy+2@gmail.com) so there are no personal identifiers (eg, email, name, and date of birth) associated with participants’ accounts. The spreadsheet containing login information and the corresponding participant’s information is stored in a secure, password-protected database. After receiving instructions and login information, participants have the option of scheduling a phone call or Zoom (Zoom Video Communications) meeting with the RA to assist with setting up their app. Figure 2 summarizes the recruitment and enrollment procedures.

### Treatment Conditions

Participants in both treatment conditions (HMP or WA) listen to prescribed guided listening on a mobile app for at least ten minutes per day (70 minutes per week) for 12 weeks. Participants download their assigned app through the Google Play (Google LLC) or Apple App (Apple Inc) Store, and participants are blinded to the condition (ie, intervention or control). Participants can complete their daily listening at any time of day. Both apps suggest a specific ordering of the audio clips, but participants can choose to listen to any audio content within the app based on their preferences—the only requirement is that they listen for at least 10 minutes per day. Participants are encouraged to turn on “push notifications” to remind them to do their daily listening, but this is not mandatory. Both apps allow participants to view their daily listening history and mark which content has already been listened to.

### Healthy Minds Program

Full details on the HMP app can be found on the web at Healthy Minds Innovations [52]. In summary, HMP is a self-guided meditation app that includes training in mindfulness through the 4 “pillars” of mental training: awareness, insight, connection, and purpose. Guided listening in each pillar trains listeners to be more focused and self-aware in the present moment (awareness), develop skills to strengthen compassionate connection with others (connection), learn about how a person’s thoughts affect their emotions (insight), and stay more grounded in one’s values and principles (purpose) [41,52]. Each pillar contains guided listening consisting of brief lessons labeled “learn” and meditations labeled “practice.” Participants are asked to complete at least 10 minutes of “practice” each day (meditations can be set for 5, 10, 15, 20, or 30 minutes) and are encouraged to listen to the “learn” content in conjunction with their practices. Participants have the choice to make any meditation “sitting” or “active” and can also select the speaker of each meditation, allowing for racial, ethnic, and gender diversity of the speaker. Our multidisciplinary team tailored the HMP app text that introduces and describes the mindfulness exercises to include language, case-based scenarios, and stressors that are common among caregivers of persons with ADRD (eg, gratitude for positive aspects of caregiving).

### Wellness App

WA is an investigator-developed time- and dose-matched control that offers daily caregiver-related guided listening consisting of knowledge-based podcasts for the 12-week intervention. If podcasts are longer than 10 minutes, caregivers are instructed to spread the listening out over multiple days to ensure that they are only listening for 10 minutes per day. All podcasts were individually selected by the study team to ensure that their content was relevant for caregivers of persons with ADRD. Examples of topics include “money and the unique needs of the older caregiver” and “how dementia affects sleep cycles.” Study staff also listened to ensure that no skills related to mindfulness or meditation were taught in any podcast to remove potential confounds. On the basis of feedback from 13 WA participants, we reviewed participant ratings of podcast quality and updated content to best meet the needs of participants in the control. For example, we replaced “overly negative” or “boring” content with more uplifting stories or engaging podcasts. The app has successfully been used as a control in multiple previous studies testing app-based meditation interventions [53,54].

### Strategies for Adherence

Regardless of group, participants are sent a text message each Monday of the intervention reminding them to listen to their daily content for at least ten minutes per day (ie, 70 minutes per week). This text message also includes a brief weekly survey with process measures (Textbox 1). Study staff members download and review participants’ listening data each Thursday. If a participant does not listen to at least 75% of their recommended listening in the past week (ie, <52.5 total minutes), a study staff member texts the participant a visual (ie, a “meme”) reminder catered toward their treatment condition (Multimedia Appendix 1). If participants do not consent to “meme” reminders, they receive a text message catered to their treatment condition instead (Multimedia Appendix 2). Participants who do not consent to receive text messages receive all reminders and surveys through email. If a participant does not listen to at least 75% of their recommended listening for 2 consecutive weeks, a study staff member calls this participant to troubleshoot potential barriers to adherence. Participants are also offered a monetary incentive for participating in the study (ie, US $20 for completing their posttest assessment and US $30 for completing their follow-up assessment, for a total of US $50).

**Secondary outcomes.**

**Demographics (pretest)**

- Age; gender; biological sex; sexual orientation; race; ethnicity; educational level; employment status; occupation; income; marital status; housing situation; languages spoken; health insurance status; zip code; importance of religion or faith; mental health history; current psychotropic and pain medication intake; overall physical health status; number of individuals to provide informal caregiving to; patient’s diagnoses; relationship to patient; duration of caregiving; number of hours of caregiving per week; and whether caregiver is living with patient

**Outcome measures (pretest, posttest, and follow-up)**

- Perceived Stress Scale-10 [55,56]: assesses perceived stress using a 5-point Likert scale
- Hospital Anxiety and Depression Scale [57]: 14-item measure to assess symptoms of emotional distress (anxiety and depression)
- Patient-Reported Outcomes Measurement Information Systems (PROMIS) Sleep Disturbance Short Form [58]: 8 items assessing sleep
- Applied Mindfulness Process Scale [59]: assesses mindfulness in 3 domains: decentering, positive emotion regulation, and negative emotion regulation
- Caregiver Reaction Scale [60]: assesses caregiver burden. We are administering the following subscales: role entrapment (4 items); role overload (4 items); mastery as a caregiver (4 items); self-mastery (4 items); workplace productivity (5 items)
- Healthy Minds Index [61]: a 17-item measure based on the Healthy Minds Program framework that assesses awareness, connection, insight, and purpose

**Process measures (weekly)**

- Emotional Distress Thermometer: scale (0-10) to assess how caregivers rate their level of distress each week: “During the past week, how would you rate your emotional distress on a scale from 0 to 10? (0=not at all; 10=extremely emotionally distressed)”
- Sleep Thermometer: scale (0-10) to assess how caregivers rate their quality of sleep: “During the past week, how would you rate your quality of sleep on a scale from 0 to 10? (0=extremely low quality; 10=extremely high quality)”
- Mindfulness Thermometer: scale (0-10) to assess how caregivers rate their level of mindfulness each week: “During the past week, how would you rate your mindfulness on a scale from 0 to 10? (0=not at all; 10=extremely mindful)”
- Stress Thermometer: scale (0-10) to assess how caregivers rate their level of stress each week: “During the past week, how would you rate your stress on a scale from 0 to 10? (0=not at all; 10=extremely stressed)”

**Other measures (post)**

- Modified Patient Global Impression of Change [62]: a 1-item questionnaire to assess the extent to which caregivers perceive the intervention’s improved functioning and symptoms: “Do you think your ability to manage stress associated with caring for your loved one with dementia is now improved, about the same, or worse, as compared to before your participation in this program?”
- Perceptions of daily meditations and podcast: we will assess caregivers’ perceptions of their app prescription by asking, “Do you think that the amount of prescribed app content was: too little, just enough, too much?”
- Perceptions of emails and text reminders: we will assess caregivers’ perceptions of email and text message reminders by asking, “Do you think that the amount of emails/texts you received was: too little, just enough, too much?”
- Perceptions of the overall study: we will assess caregivers’ perceptions of the study by asking, “Do you have any feedback about your experience in this program (content of the program, content of the text message reminders, etc) that you would like to share?”
- Perceptions of prompts and contacts in low adherence situations: We will assess caregivers’ perceptions of the number of prompts and contact in situations where they were not adhering to their practice by asking, “Do you think that the amount of prompts you received when you struggled with your practice was: too little, just enough, too much?”

### Feasibility Markers

Table 2 outlines the a priori set benchmarks and criteria that are consistent with guidelines for early feasibility studies [63]. We will assess the feasibility (acceptability and demand markers) of both mobile app treatment conditions and participants’ perceptions of their involvement in the study.

**Table 2:** Primary outcomes.

Benchmarks and markers			Description		Criteria
**Acceptability benchmarks**					
	Feasibility of recruitment	We will assess the number of caregivers enrolled from the number of caregivers who were eligible		80 caregivers will be recruited in 6 months; ≥70% of eligible caregivers will enroll	
	Treatment satisfaction	We will use the Client Satisfaction Questionnaire [64] to assess caregivers’ satisfaction with the intervention		At least 70% of caregivers will have scores on the Client Satisfaction Scale over and above the midpoint in both the HMP^a^ and WA^b^ groups	
	Credibility and expectancy	We will use the Credibility and Expectancy Questionnaire [65] to assess caregivers’ perceptions that the treatment will work after learning about the study.		At least 70% of caregivers will have scores on the Credibility and Expectancy Scale above the midpoint in both the HMP and WA groups.	
**Demand benchmarks**					
	Adherence to treatment	We will assess data from the HMP app and the WA to measure adherence to the intervention and the control conditions		≥70% of caregivers will complete ≥75% of weekly prescribed minutes	
	Dropout	We will assess the number of caregivers who complete the study from the number of caregivers who started the study		>70% of caregivers who start the study will complete posttest assessments; ≤30% attrition	
**Data collection benchmarks**					
	Feasibility of weekly REDCap^c^ measures	We will assess the feasibility of the weekly REDCap measures sent to patients		≥70% caregivers will answer ≥75% of weekly REDCap measures	
	Feasibility of quantitative measures	We will assess the feasibility of the quantitative measures sent to caregivers at baseline, postintervention, and follow-up		No questionnaires fully missing in ≥25% caregivers	

^a^HMP: Healthy Minds Program.

^b^WA: Wellness App.

^c^REDCap: Research Electronic Data Capture

### Quantitative Assessments

The self-report measures and assessment domains were selected consistent with the purpose of the study [66]. Demographics are measured pretest, outcome measures are given during the main assessment points (pretest, posttest, and follow-up), and process measures are administered weekly. Textbox 1 provides a brief description of all self-reported assessments.

### Data Analysis

We will assess the acceptability and demand of the intervention in line with Bowen and colleagues’ standards for feasibility studies and components of the Behavioral Change Consortium’s Treatment Fidelity model [63,67]. Specifically, we will assess the acceptability, credibility, satisfaction, and perceived appropriateness and usefulness of the daily prescribed app content. Benchmarks and measurements for each feasibility construct are listed in Table 2. One-sided 95% CI will be calculated for each proportion to evaluate feasibility benchmarks. Depending on the nature of the estimate or comparison, exact binomial tests, Fisher exact tests, 2-tailed *t* tests, or correlations may be used to summarize the data and test for statistical significance. HMP and WA user data (ie, what content users listened to, when, and for how long) are collected automatically from users.

Consistent with guidelines for early feasibility studies, our analysis will not assess efficacy, as we are not powered to do so [68]. However, we will estimate between-group differences in changes from baseline to posttest (12 weeks) to follow-up (20 weeks) as proof of concept that the intervention shows promise with the intended population [63]. Variance components and effect sizes will be estimated for each outcome. We will use variance estimates from repeated-measures analysis of data on Perceived Stress Scale-10 to guide the design and power calculations for a future trial testing efficacy [69,70]. We will compute the effect sizes of change in these scores to cautiously guide future work. Proof of concept will be demonstrated if the decrease in caregiver stress (Perceived Stress Scale-10, the primary outcome in future trials) in the intervention group from baseline to posttest is greater than in the WA group, with at least small to moderate effect sizes favoring HMP (between-group test of superiority).

## Results

The trial is ongoing. As of June 2023, we had enrolled 27 caregivers (14 HMP and 13 WA). We aim to recruit 80 caregivers in total. Table 3 details the select demographics of enrolled participants.

**Table 3:** Demographics for enrolled participants.

Variable			Participants, n (%)
**Gender**			
	Women	24 (89)	
	Men	3 (11)	
**Sexual orientation**			
	Heterosexual	26 (96)	
	Lesbian	1 (4)	
**Race**			
	White	24 (89)	
	Black or African American	2 (7)	
	More than 1 race	1 (4)	
**Ethnicity**			
	Not Hispanic or Latino or Latina	27 (100)	
**Income (US $)**			
	Chose not to answer	4 (15)	
	15,000-29,000	3 (11)	
	30,000-49,000	2 (7)	
	50,000-69,000	1 (4)	
	70,000-99,000	9 (33)	
	>100,000	8 (30)	
**Relationship to care recipient**			
	Adult child (caring for parent)	12 (44)	
	Spouse	11 (41)	
	Friend or nonkin	2 (7)	
	Sibling	1 (4)	
	Other	1 (4)	

## Discussion

### Scientific Contribution

The intervention at present is catered to caregivers’ interest in using mobile apps for stress management and health information [35] including learning mindfulness meditation for stress management [36-39]. This modality addresses barriers to caregiver participation (eg, in-person, synchronous format, time commitment, and cost) for a more feasible, scalable, and cost-effective intervention to reduce stress among this high-need population. The HMP app adapted for caregivers of persons with ADRD seeks to address this clinical gap by testing a no-cost mobile app to reduce stress, emotional distress, and sleep difficulties [40]. This protocol describes an entirely remote, single-blind feasibility, proof-of-concept RCT of HMP versus a time- and dose-matched educational control (WA) in stressed caregivers of persons with ADRD. Our protocol also introduces novel strategies for addressing concerns in mobile app–based RCTs with caregivers, including a protocol for managing “imposter” participants and using “memes” for promoting adherence and engagement.

### Limitations

There are important limitations to note. First, our initial recruitment efforts have yielded limited diversity in terms of enrolling caregivers from racial and ethnic minoritized (89% White, 100% non-Hispanic) as well as sexual and gender minoritized backgrounds (96% heterosexual, 100% cisgender). We are now modifying recruitment efforts to attempt to recruit a more representative sample (eg, sharing recruitment flyers with community organizations that serve minoritized older adults and caregivers, and developing Instagram advertisements that target geographically diverse populations). Second, we are delivering this program to English-speaking caregivers only. In the future, we hope to offer HMP in different languages. Third, the fully remote nature of the study increases the likelihood of enrolling imposter participants that are not truly caregivers of persons with ADRD but rather seek to participate for financial benefits; see the Methods section for a detailed plan of how we are addressing this issue. Lastly, it is possible that participants may be unblinded if they carry out web searches about our research.

### Conclusions

In summary, we are currently testing the feasibility and proof of concept of HMP versus a time- and dose-matched health education control (WA). Between February and June 2023, we had 27 caregivers enrolled, and we aim to recruit 80 caregivers in total. At the conclusion of this trial, we will examine whether HMP and WA meet feasibility (acceptability and demand) benchmarks, as well as if HMP demonstrates proof of concept in improving stress, emotional distress, sleep, and mindfulness above WA in stressed caregivers of persons with ADRD. Our findings will inform a future, fully powered RCT of HMP versus WA. This work has the potential to pave the way for feasible, scalable, and cost-effective interventions for caregivers of persons with ADRD.

## Appendix

Multimedia Appendix 1 Visual (ie, “meme”) reminders for low-adherence situations.

Multimedia Appendix 2 Engaging text message reminders for low-adherence situations.

Multimedia Appendix 3 Peer-review reports from Clinical Management in General Care Settings Study Section, Healthcare Delivery and Methodologies Integrated Review Group, Center for Scientific Review, CMGC, NIH (USA).

## Abbreviations

ADRD: Alzheimer disease and related dementias
FAST: Functional Assessment Staging Tool
HMP: Healthy Minds Program
MBI: mindfulness-based intervention
NIH: National Institutes of Health
RA: research assistant
RCT: randomized controlled trial
REDCap: Research Electronic Data Capture
SPMSQ: Short Portable Mental Status Questionnaire
WA: Wellness App

## Data Availability

The data sets generated during this study are not publicly available due to the ongoing nature of the trial but will be available from the corresponding author on reasonable request upon conclusion of the study.
