# Supplementary figures and images for: Mindfulness-Based App to Reduce Stress in Caregivers of Persons With Alzheimer Disease and Related Dementias: Protocol for a Single-Blind Feasibility Proof-of-Concept Randomized Controlled Trial
Source: JMIR Res Protoc. 2023 Oct 13;12:e50108. doi: 10.2196/50108 (PMC10612010; doi:10.2196/50108)

**Multimedia Appendix 1. Visual (i.e., “meme”) reminders for low-adherence situations.**

**
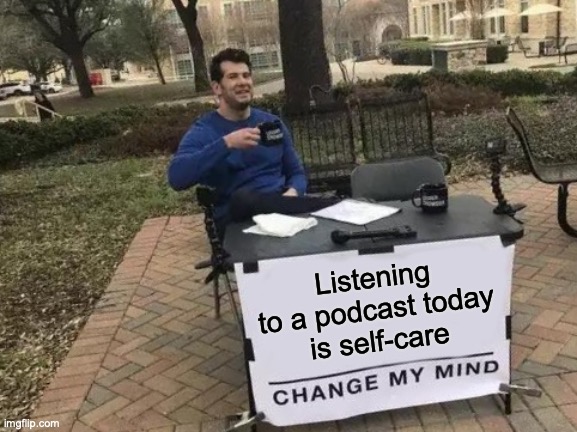

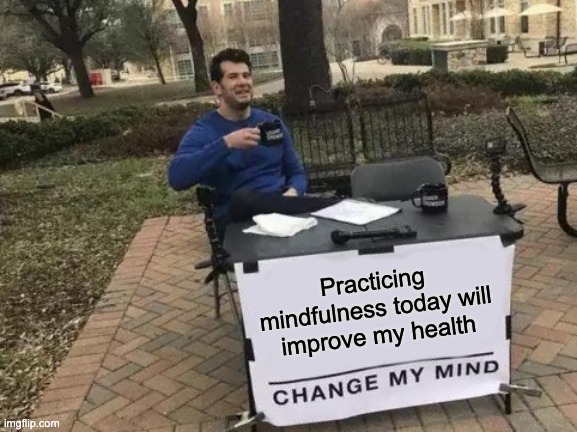
**

**
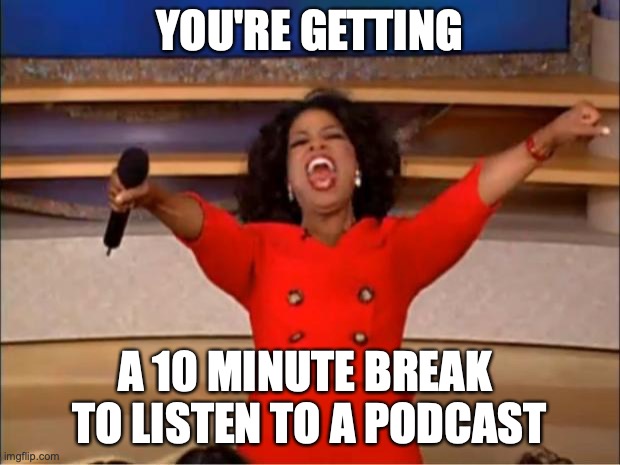

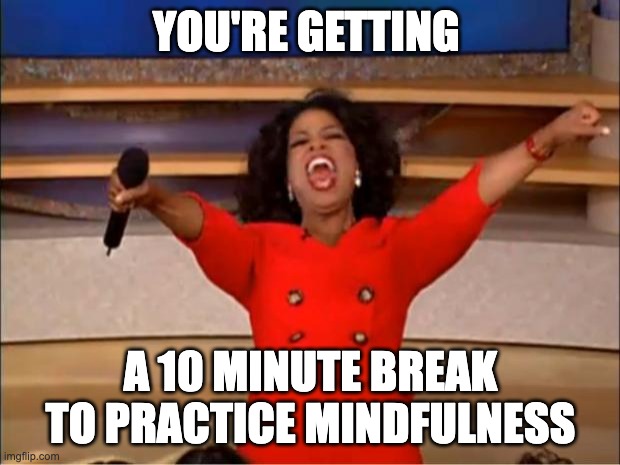
**

**
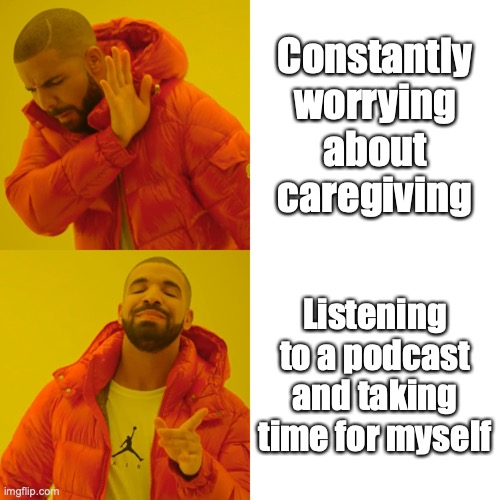
**

**
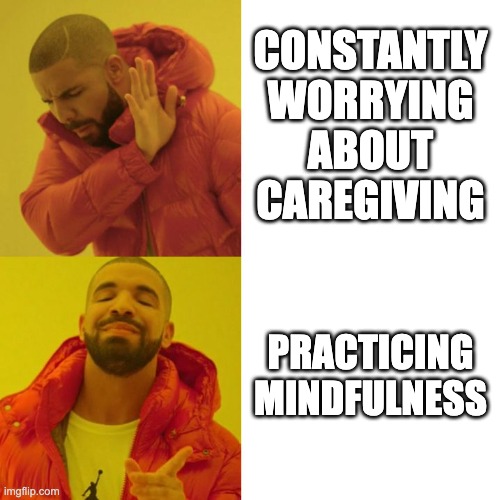
**

**
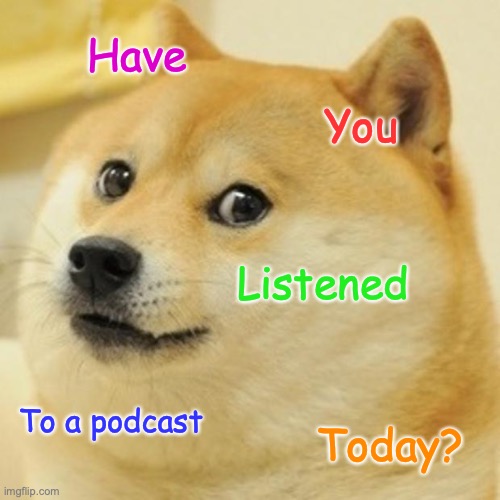

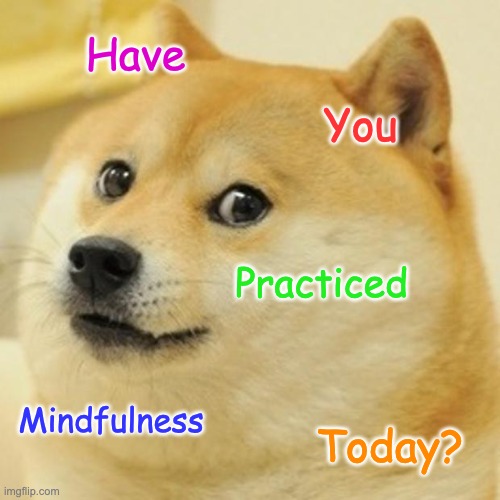
**

**
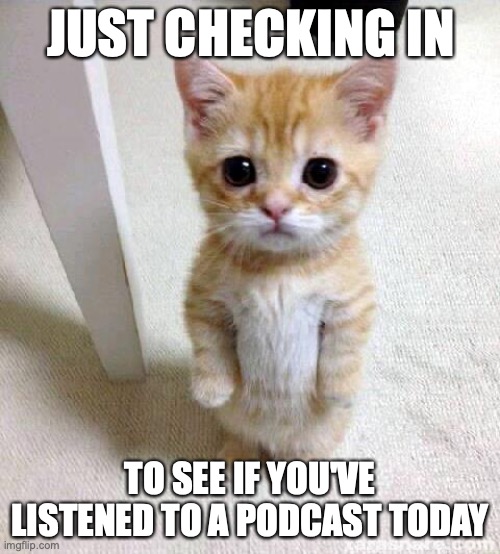
**

**
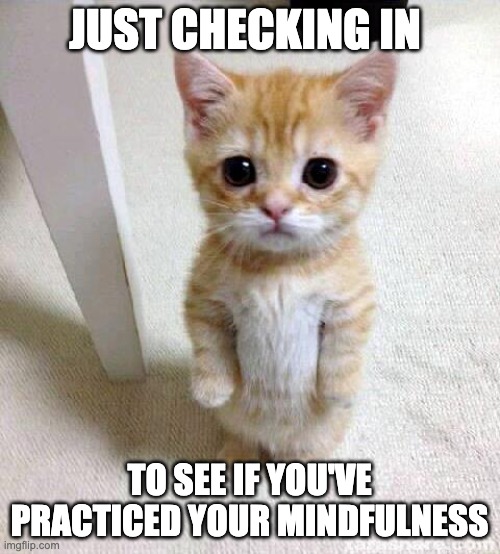
**

**
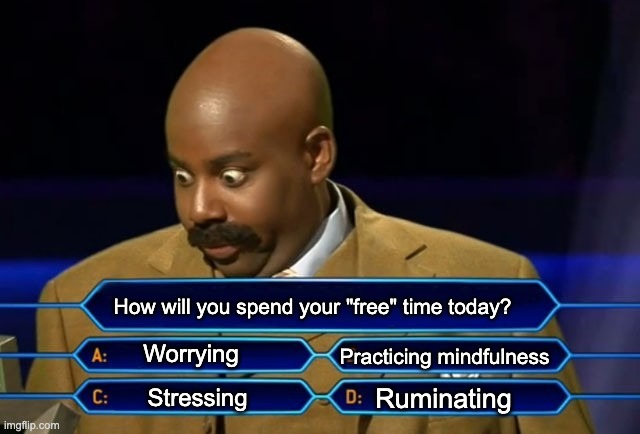
**


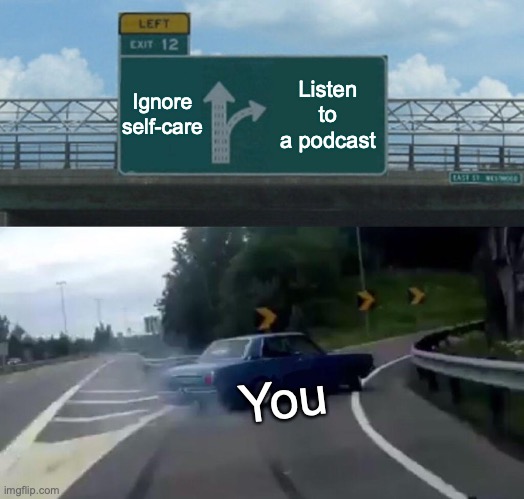

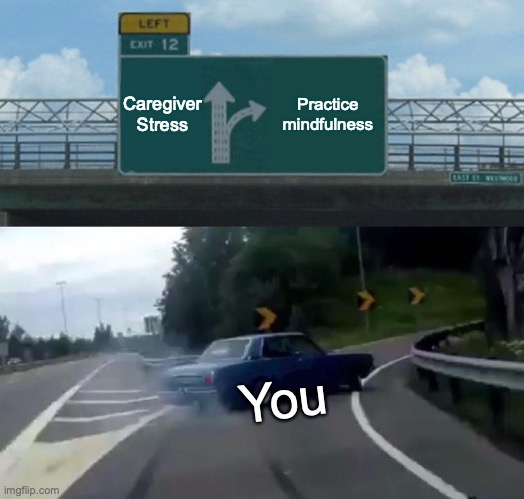


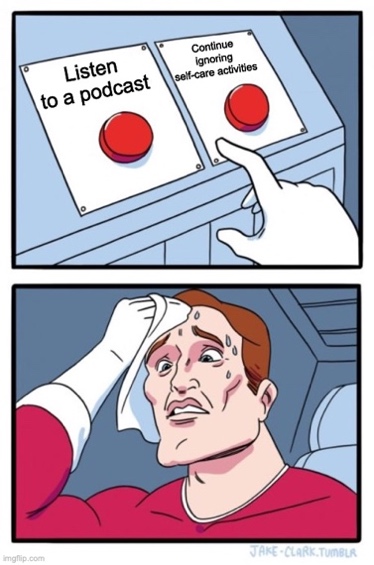


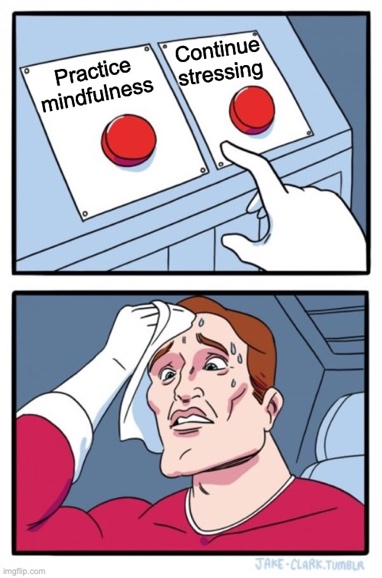


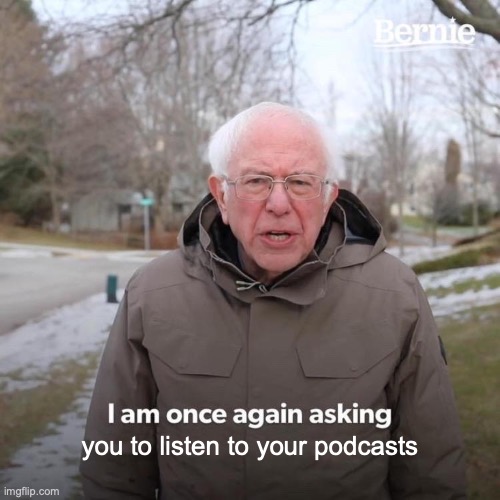


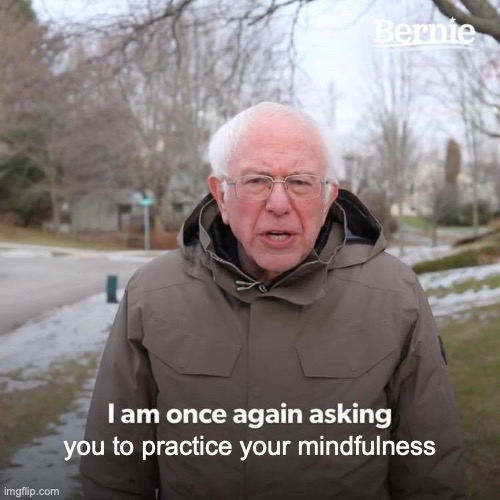


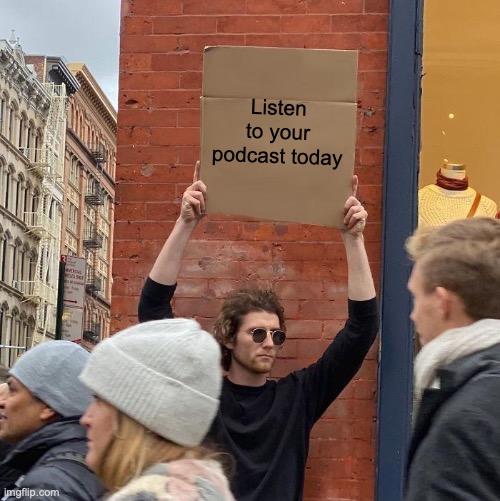

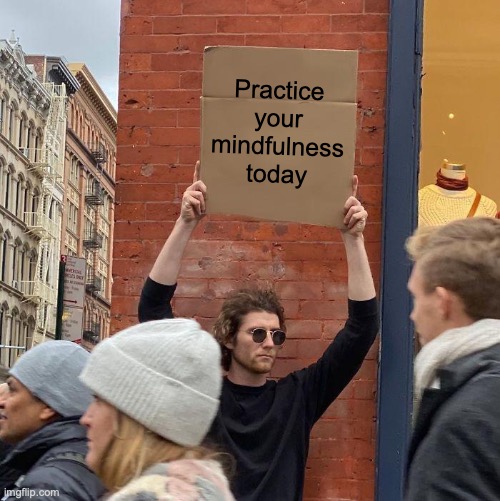

Supplement: Multimedia Appendix 1 [file resprot_v12i1e50108_app1.docx]
