# Supplementary material for: Mindfulness-Based App to Reduce Stress in Caregivers of Persons With Alzheimer Disease and Related Dementias: Protocol for a Single-Blind Feasibility Proof-of-Concept Randomized Controlled Trial
Source: JMIR Res Protoc. 2023 Oct 13;12:e50108. doi: 10.2196/50108 (PMC10612010; doi:10.2196/50108)
Supplement: Multimedia Appendix 2 [file resprot_v12i1e50108_app2.docx]

**Multimedia Appendix 2. Engaging text message reminders in low-adherence situations.**

Healthy Minds Program (HMP)

1. Are you doing that thing… that thing where you put other things above your mental health again? It’s ok! Today, try practicing mindfulness.
2. Having trouble getting your meditations in? Winston Churchill wanted you to know: *"Success is not final, failure is not fatal: it is the courage to continue that counts."* Try to meditate for 10 minutes today!
3. Every time you practice mindfulness an angel gets its wings*

*This claim is untested… for now. Make sure to practice 10 minutes/day and see what happens!

1. If you can read this message, you can practice mindfulness! But if you are reading this message, you didn’t practice mindfulness. That’s OK! Nelson Mandela once said: *“The greatest glory in living lies not in never failing, but in rising every time we fail.”*  Try to practice for ten minutes today!
2. Have you given your M.O.M. (Mindfulness or Mediation) any attention today? 🧘🏻‍♀️🧘🏽🧘🏼‍♂️
3. It’s normal to struggle with practicing – Taylor Swift doesn’t want you to beat yourself up about it. She wants you to read this quote and feel inspired to try meditating again:
   1. *"I just want you to know that if you are out there and you are being really hard on yourself right now for something that has happened ... it's normal…Please be kind to yourselves and stand up for yourself, please."*
4. Stress can wait, meditate! With just 10 minutes per day you’ll be on your way to less stress and more calm 😌 .
5. J Lo wanted to tell you: “*You get what you give. What you put into things is what you get out of them.”* The more you put into practicing your mindfulness, the more improvements in stress you will see. Aim for ten minutes per day, you got this!
6. It’s never too late to get back on the meditation train – aim for those 10 minutes per day! “*The greatest accomplishment is not in never falling, but in rising again after you fall*.”— Vince Lombardi
7. Did you know, mindfulness contains the word ‘unmissed’? I mean it’s right there in the title, don’t miss your mindfulness: “*Believe you can and you are halfway there*” – Theodore Roosevelt”
8. Look, we get it, there’s a million things to do. Mindfulness doesn’t have to be number 1, but top 50? Ok, maybe not top 50, but at least higher than television reruns! You’ve already seen them, you know what happens!
9. Having trouble meditating? Aim for just 10 minutes per day. Dwayne “The Rock” Johnson wanted to share his motto with you: *"Wake up determined, go to bed satisfied."*
10. Less sitcom, more sit calm (and meditate – for 10 minutes every day).
11. Having trouble practicing? There’s still time to get back on the wagon! “*We may encounter many defeats but we must not be defeated.*”– Maya Angelou
12. *“I love that you are the last person I want to talk to before I go to sleep at night."*

-Harry, from the movie When Harry Met Sally

He was talking about his mindfulness app, you know ;)

Wellness App (WA)

1. Are you doing that thing… that thing where you put other things above your personal growth again? It’s ok! Today, try listening to a podcast.
2. Having trouble listening to your podcasts? Winston Churchill wanted you to know: *"Success is not final, failure is not fatal: it is the courage to continue that counts."* Try to listen for 10 minutes today!
3. Every time you listen to a podcast an angel gets its wings*

*This claim is untested… for now. Make sure to listen for 10 minutes/day and see what happens!

1. If you can read this message, you can listen to your podcasts! But if you are reading this message, you didn’t listen to your podcasts. That’s OK! Nelson Mandela once said: *“The greatest glory in living lies not in never failing, but in rising every time we fail.”*
2. It’s normal to struggle with a new habit – Taylor Swift doesn’t want you to beat yourself up. She wants you to read this quote and feel inspired to listen to your podcasts instead:
   1. *"I just want you to know that if you are out there and you are being really hard on yourself right now for something that has happened ... it's normal…Please be kind to yourselves and stand up for yourself, please."*
3. It’s never too late to get back on the podcast train – aim for those 10 minutes per day! *“The greatest accomplishment is not in never falling, but in rising again after you fall.”*— Vince Lombardi
4. J Lo wanted to tell you: “*You get what you give. What you put into things is what you get out of them.”* Remember to take time for yourself. Aim to listen for ten minutes per day, you got this!
5. Having trouble listening to your podcasts? Theodore Roosevelt has a message for you: *“Believe you can and you are halfway there”*
6. Look, we get it, there’s a million things to do. Listening to a podcast doesn’t have to be number 1, but top 50? Ok, maybe not top 50, but at least higher than television reruns! You’ve already seen them, you know what happens!
7. Having trouble listening to your podcasts? Aim for just 10 minutes per day. Dwayne “The Rock” Johnson wanted to share his motto with you: *"Wake up determined, go to bed satisfied."*
8. Having trouble listening? There’s still time to get back on the wagon! “*We may encounter many defeats but we must not be defeated.*”– Maya Angelou
9. *“I love that you are the last person I want to talk to before I go to sleep at night."*

-Harry, from the movie When Harry Met Sally

He was talking about his podcast app, you know ;)
