# Supplementary material for: Mindfulness-Based App to Reduce Stress in Caregivers of Persons With Alzheimer Disease and Related Dementias: Protocol for a Single-Blind Feasibility Proof-of-Concept Randomized Controlled Trial
Source: JMIR Res Protoc. 2023 Oct 13;12:e50108. doi: 10.2196/50108 (PMC10612010; doi:10.2196/50108)
Supplement: Multimedia Appendix 3 [file resprot_v12i1e50108_app3.pdf]

SUMMARY STATEMENT
(Privileged Communication)

PROGRAM CONTACT:
Dr Elizabeth Necka
301-496-3136
liz.necka@nih.gov

Release Date: 03/01/2022
Revised Date:

Application Number: 1 R21 AG075187-01A1
Formerly: 1R21AG075187-01

Principal Investigators (Listed Alphabetically):

HUBERTY, JENNIFER LYNNE
VRANCEANU, ANA-MARIA (Contact)

Applicant Organization: MASSACHUSETTS GENERAL HOSPITAL

Review Group: CMGC
Clinical Management in General Care Settings Study Section

Meeting Date: 02/07/2022
Council: MAY 2022
Requested Start: 07/01/2022

RFA/PA: PA20-194
PCC: 2BBCHEN
Dual IC(s): NR, MH

Project Title: Using Mobile Technology to Reduce Stress in Caregivers of Persons with Dementia. A Scalable Solution to A Growing Problem
SRG Action: Impact Score:26 Percentile:10 +
Next Steps: Visit https://grants.nih.gov/grants/next_steps.htm
Human Subjects: 30-Human subjects involved - Certified, no SRG concerns
Animal Subjects: 10-No live vertebrate animals involved for competing appl.
Gender: 1A-Both genders, scientifically acceptable
Minority: 1A-Minorities and non-minorities, scientifically acceptable
Age: 3A-No children included, scientifically acceptable

Project Year	Direct Costs Requested	Estimated Total Cost
1	150,000	236,028
2	125,000	196,690
TOTAL	275,000	432,718

ADMINISTRATIVE BUDGET NOTE: The budget shown is the requested budget and has not been adjusted to reflect any recommendations made by reviewers. If an award is planned, the costs will be calculated by Institute grants management staff based on the recommendations outlined below in the COMMITTEE BUDGET RECOMMENDATIONS section.

1R21AG075187-01A1 Vranceanu, Ana-Maria

RESUME AND SUMMARY OF DISCUSSION: This application proposes to test the feasibility and proof of concept of a mobile health intervention teaching meditation and stress reduction techniques for caregivers of people living with Alzheimer's disease and related dementias (ADRD). This resubmission is responsive to prior reviews including addressing concerns about tailoring prompts for caregiving and providing data about caregiver interest in the mobile health application. Reviewers disagreed on the significance of the application, although some reviewers articulated that a mobile health application targeting caregiver stress through a brief dose of a mindfulness program is highly significant, other reviewers argued that the dose is too low and the costs for the mobile application are not addressed which could potentially influence scalability. The project is led by a strong research team with complementary expertise and a prior history of collaboration. The mHealth mindfulness intervention for caregivers is innovative as it alleviates the need for a coach and attendance at a specific course. Strengths in the approach include a YouTube video to train participants on using the mobile application as well as the availability of live help. Text prompts remind participants to engage with the app and smart phones and data plans will be provided to participants if needed. Reviewers weighed these strengths and weaknesses differently, and overall, this is an innovative application led by a strong research team that will have a high impact on stress reduction among caregivers of people living with Alzheimer's disease and related dementias.

DESCRIPTION (provided by applicant): Using the NIA 2 year R21 grant mechanisms, the proposed study will examine the feasibility (markers of acceptability and demand), and proof of concept of the consumer based mobile mindfulness application Calm in decreasing stress, emotional distress and sleep dysfunction in heterogeneous, geographically diverse, stress caregivers of individuals with ADRD. Calm teaches fundamentals of mindfulness and uses evidence based behavioral principles to engage caregivers in sustained practice (>10 min/day) with minimal time commitment, when it is convenient for them, without the need of travel or of a trained provider. To achieve our aims, we will conduct a single blind, pilot, feasibility, proof of concept RCT of CALM (12 weeks, > 10 minutes daily practice) versus an attention placebo time and dose matched podcast control (POD; 12 weeks, >10 minutes daily listening). Assessments will be conducted at baseline, post program (12 weeks later) and at 20 weeks follow-up. Caregivers will also complete weekly assessments. Our team is well positioned to conduct this study with specific experience with mobile applications including Calm; intervention development, feasibility testing and clinical trials; ADRD caregivers; recruitment of national, geographically diverse samples; R21 grant proposals. At the end of the 2-year period of this R21 we will have clear evidence of the feasibility of study procedures, randomization, acceptability, credibility, adherence, ability to retain and recruit as well as its proof of concepts in reducing stress in caregivers of people with ADRD. Results from this R21 study will be used to apply for funding to conduct a fully powered efficacy RCT, if set benchmarks are met (see Table in the Research Strategy), through an R01. The goal is dissemination and implementation of Calm to reduce stress, emotional dysfunction, and sleep difficulties among all caregivers of individuals with ADRD.

PUBLIC HEALTH RELEVANCE: By reducing stress in caregivers of ADRD, the proposed work has the potential to drastically improve emotional and physical health in not only caregivers, but also the people they care for. Calm teaches meditation, an evidence-based stress reduction technique without the burden associated with traditional in person or virtual meditation-based interventions. As such, there is tremendous opportunity for scalability and improved care for ADRD caregivers across the world.

CRITIQUE 1

Significance: 4
Investigator(s): 4
Innovation: 4
Approach: 5
Environment: 3

Overall Impact: The proposed R21 resubmission will test feasibility and proof of concept of CALM, a mindfulness app that intervention group dementia caregivers will use for 10-12 minutes daily over 12 weeks – in comparison to attention control caregivers who will listen to 10-12 minutes daily of a pod cast. Data collected at baseline, post intervention and 20-week follow-up will be compared for change in measures of stress, emotional dysfunction, and sleep difficulties and participant engagement will also be compared. This resubmission has responded to the prior critique issues. The problem is significant with growing need for informal caregivers for PWD who experience stress and resulting negative caregiving outcomes and positive stress relieve experienced with mindfulness interventions in other populations. Significance is reduced by the low dose of the intervention's questionable ability to counteract multiple and diverse stresses encountered by caregivers. Acceptability to underserved populations and expense for app use are not addressed in consideration of scalability. The investigative team has requisite skills and expertise to conduct the study. However, the statistician effort is likely inadequate to complete analyses at 1-2.5% effort. The environment is strong with resources to support the study and letters of support provided. Receiving the intervention via an app is somewhat innovative although the app has been commercially available to the public for some time. Costs for the app are not detailed. The approach includes a number of strengths and weaknesses. Score driving strengths include the detail in intervention description, and additions of You Tube app training for participants, text prompts for engagement, and provision of smart devices and data plans if needed. Data on engagement will be collected electronically, benchmarks for success are delineated, analysis will be blinded and the study is well grounded in theory. Weaknesses include inadequate addition of engineering support; limiting caregivers to those with high levels of stress; heterogeneity in caregiver amount and type of caregiving assistance provided; heterogeneity in PWD type of dementia, severity, and presence of BPSDs; and need for potential participants to actively seek out enrollment. Due to the number of study strengths and weaknesses, the overall potential impact of this study is moderately high.

1. Significance:

Strengths

- Stress is a significant problem and predictor of negative outcomes for the growing number of caregivers for PWD.
- An asynchronous, accessible stress reduction intervention overcomes multiple barriers and could provide significant and needed benefits for dementia caregivers.
- Strong background on stress reduction and associated benefits of mindfulness is provided.

Weaknesses

- Although >10 minutes daily mindfulness practice may reduce stress somewhat, it is a relatively low dose of intervention to overcome numerous stressful experiences and negative responses for caregivers.
- Unclear whether mindfulness app is acceptable to health disparate populations.
- Calm app use would scalable and relatively inexpensive

- There is no need to establish feasibility for recruitment based on investigators reported prior study success with this population.

2. Investigator(s):

Strengths

- MPIs have expertise in complementary intervention development and testing using technology-based interventions targeting dementia caregivers.
- Team are established collaborators.

Weaknesses

- Extremely low effort for biostatistician 1% year 1, 2.5% year 2, may be inadequate to conduct analyses and related activities.

3. Innovation:

Strengths

- Overcoming need for a coach, attendance at a set time and place, to receive a caregiver intervention.

Weaknesses

- Caregivers currently have access to the commercially available app.

4. Approach:

Strengths

- Addition of You Tube training in app use with availability of live help.
- Addition of text prompts to remind participants to engage.
- Addition of providing smart phones or other devices and data plan if participants do not have these.
- Study is grounded in a theoretical framework.
- Participant engagement in intervention and control activities will be electronically monitored.
- Blinding of data analyzers.
- Detailed inclusion criteria.
- Realistic estimates of attrition.
- Details about the content of the intervention are provided.
- Benchmarks for success.

Weaknesses

- Participant completion of weekly stress, distress, and satisfaction may be burdensome and likely will take longer than 10-20 seconds total.
- Additional 3 hours of engineering support each year is likely inadequate to make a difference.
- Inclusion of only those caregivers experiencing high levels of stress is not representative and will bias sample.

- Heterogeneity of PWD (type of dementia, stage, presence of BPSD) population will confound findings.
- Heterogeneity in caregivers (relationship, time and specific duties) will confound findings.
- There is no need to establish feasibility for recruitment based on investigators reported prior study success with this population.
- Proposal should primarily focus on this study versus future R01 RCT.
- Potential participants must actively seek out enrollment online.
- Although the National Alliance for Caregiving identifies racial and ethnic distribution, caregivers of different backgrounds may be more or less likely to participate so that diversity cannot be assured.

5. Environment:

Strengths

- Mass General Hospital has resources to support the study conduct.
- Partnership with National alliance of Caregiving.

Weaknesses

- PIs involvement as board member for commercial CALM app could present conflict of interest.

Study Timeline:

Strengths

- Detailed timeline provided.

Weaknesses

- None noted.

Protections for Human Subjects:

Acceptable Risks and/or Adequate Protections

- Adequate plan

Data and Safety Monitoring Plan (Applicable for Clinical Trials Only):

Acceptable

- o Independent safety monitor.

Inclusion Plans:

- Sex/Gender: Distribution justified scientifically
- Race/Ethnicity: Distribution justified scientifically
- For NIH-Defined Phase III trials, Plans for valid design and analysis: Not applicable
- Inclusion/Exclusion Based on Age: Distribution justified scientifically
- Anticipate sample will reflect National Alliance for Caregiving membership.

Vertebrate Animals:
Not Applicable (No Vertebrate Animals)

Biohazards:
Not Applicable (No Biohazards)

Resubmission:

- Somewhat responsive

Resource Sharing Plans:
Acceptable

Budget and Period of Support:
Recommend as Requested

CRITIQUE 2

Significance: 1
Investigator(s): 1
Innovation: 1
Approach: 3
Environment: 1

Overall Impact: This R21 proposal will test the widely available Calm app with caregivers of patients with ADRD. This resubmission is highly responsive to reviewer comments. The pilot study is carefully designed with appropriate assessment of feasibility, acceptability and preliminary effects. One weakness is the lack of consideration for cultural differences with response to interest in or openness to meditation as an approach to reducing stress.

1. Significance:
Strengths

- The need is great for interventions to reduce caregiver stress in ADRD.
- There is prior evidence that mindfulness programs can reduce stress.
- The revision provides evidence that ADRD caregivers are open to using a mobile app.

Weaknesses

- None

2. Investigator(s):
Strengths

- Co-PI Dr. Vranceanu has expertise in app development and mindfulness based interventions.
- Co-PI Dr. Huberty is the scientific director at Calm
- Dr. Mace adds expertise in dementia care.
- The team has active collaborations

Weaknesses

- None

3. Innovation:

Strengths

- Rigorous study of a mindfulness app for ADRD caregivers is innovative.

Weaknesses

- None

4. Approach:

Strengths

- The attention control group is appropriate for the study.
- The study will enroll caregivers with high baseline stress.
- The study pairs the Calm app with automated messages that are tailored for caregivers. This allows the use of a widely available app without modification.

Weaknesses

- Using REDcap for all data collection may lead to higher loss to follow-up. Are there any alternatives for participants who do not respond to online surveys?
- There is no consideration of cultural or racial differences in openness to meditation in the significance or stud design.

5. Environment:

Strengths

- MGH has the resources needed to carry out the proposed project.
- They are partnering with the Calm company in this work.

Weaknesses

- None

Study Timeline:

Strengths

- The timeline is clear and appropriate.

Weaknesses

- None

Protections for Human Subjects:

Acceptable Risks and/or Adequate Protections

- Protections are acceptable.

Data and Safety Monitoring Plan (Applicable for Clinical Trials Only):

Acceptable

- Will have a data safety monitor.

Inclusion Plans:

- Sex/Gender: Distribution justified scientifically
- Race/Ethnicity: Distribution justified scientifically
- For NIH-Defined Phase III trials, Plans for valid design and analysis: Not applicable
- Inclusion/Exclusion Based on Age: Distribution justified scientifically
- All plans are appropriate.

Vertebrate Animals:

Not Applicable (No Vertebrate Animals)

Biohazards:

Not Applicable (No Biohazards)

Resubmission:

- The revised proposal is highly responsive to critiques. They address concerns about the need for tailoring by using prompts related to caregiving. They provide additional data about caregiver interest in a mobile application. They have clarified several additional points.

Resource Sharing Plans:

Not Applicable (No Relevant Resources)

Budget and Period of Support:

Recommend as Requested

CRITIQUE 3

Significance: 3
Investigator(s): 1
Innovation: 2
Approach: 2
Environment: 1

Overall Impact: The proposed research is to evaluate the feasibility/acceptability and preliminary efficacy of a mindfulness intervention delivered via smartphone to help informal caregivers of individuals with ADRD manage stress. The application has many strengths, including well justified scientific premise, a strong investigative team, preliminary data, and well thought-out research plans. My minor concerns include a lack of novelty in research questions and unspecified mechanisms that are responsible for intervention effects, which would be an important scientific question for future development of similar apps as opposed to testing whether one particular app is efficacious or not.

1. Significance:

Strengths

- Addressing dementia caregivers' stress management is a significant public health concern.
- Among numerous apps, why the team chose Calm is clearly described.

Weaknesses

- However, the extent to which the team reviewed existing commercial apps is not described. In other words, is Calm the only app that uses evidence-based strategies or are there many other alternates.
- The active/core theoretical elements of the intervention that may be responsible for changes in behaviors and health outcomes could be delineated.
- Given that the intervention is a commercial app, the extent to which the app can be modified (as needed) after the study is unclear.

2. Investigator(s):

Strengths

- The team of investigators is very strong with appropriate expertise and the history of working together.

Weaknesses

- None

3. Innovation:

Strengths

- It is claimed that none of the existing mindfulness apps has been tested for efficacy.

Weaknesses

- Mindfulness interventions to manage and reduce stress have been tested, use of smartphones as an intervention medium has become ubiquitous, and in fact, there are mobile phone based interventions tested in an RCT (based on a recent systematic metareview of meta-analyses; though those studies have many issues), and stress management interventions targeting caregivers of dementia patients (either stand alone or as part of caregiver support interventions) have been tested. It is unclear what novel questions are asked in this application.

4. Approach:

Strengths

- A strong and thoughtful RCT design with an appropriate attention placebo control and inclusion criteria.
- Clear benchmarks to determine feasibility/acceptability.
- Although not required, preliminary data to support the proposed research.
- Attention to the control condition to mimic the intervention.
- Attention to fidelity and adherence

Weaknesses

- The rationale for 3-month follow-up assessment is unclear; the preliminary work doesn't state follow-up times in the three studies.
- The Y/N questions related to acceptability will likely yield limited feedback from the participants.
- No strategies to maximize minority recruitment and retention are employed.
- Sex as a biological variable is not addressed.

5. Environment:

Strengths

- Both MGH and ASU provide excellent research infrastructures.
- The infrastructure to be used for the proposed study's recruitment

Weaknesses

- None noted by reviewer

Study Timeline:

Strengths

- Reasonable and feasible

Weaknesses

- None noted by reviewer

Protections for Human Subjects:

Acceptable Risks and/or Adequate Protections

Data and Safety Monitoring Plan (Applicable for Clinical Trials Only):

Acceptable

Inclusion Plans:

- Sex/Gender: Distribution justified scientifically
- Race/Ethnicity: Distribution justified scientifically
- For NIH-Defined Phase III trials, Plans for valid design and analysis:
- Inclusion/Exclusion Based on Age: Distribution justified scientifically

Vertebrate Animals:
Not Applicable (No Vertebrate Animals)

Biohazards:
Not Applicable (No Biohazards)

Resubmission:

- The revised application is responsive to the previous criticisms.

Resource Sharing Plans:

Budget and Period of Support:
Recommend as Requested

THE FOLLOWING SECTIONS WERE PREPARED BY THE SCIENTIFIC REVIEW OFFICER TO SUMMARIZE THE OUTCOME OF DISCUSSIONS OF THE REVIEW COMMITTEE, OR REVIEWERS' WRITTEN CRITIQUES, ON THE FOLLOWING ISSUES:

PROTECTION OF HUMAN SUBJECTS: ACCEPTABLE

INCLUSION OF WOMEN PLAN: ACCEPTABLE

INCLUSION OF MINORITIES PLAN: ACCEPTABLE

INCLUSION ACROSS THE LIFESPAN: ACCEPTABLE

COMMITTEE BUDGET RECOMMENDATIONS: The budget was recommended as requested.

Footnotes for 1 R21 AG075187-01A1; PI Name: Vranceanu, Ana-Maria

+ Derived from the range of percentile values calculated for the study section that reviewed this application.

NIH has modified its policy regarding the receipt of resubmissions (amended applications).See Guide Notice NOT-OD-18-197 at https://grants.nih.gov/grants/guide/notice-files/NOT-OD-18-197.html. The impact/priority score is calculated after discussion of an application by averaging the overall scores (1-9) given by all voting reviewers on the committee and multiplying by 10. The criterion scores are submitted prior to the meeting by the individual reviewers assigned to an application, and are not discussed specifically at the review meeting or calculated into the overall impact score. Some applications also receive a percentile

ranking. For details on the review process, see http://grants.nih.gov/grants/peer_review_process.htm#scoring.

MEETING ROSTER

Clinical Management in General Care Settings Study Section
Healthcare Delivery and Methodologies Integrated Review Group
CENTER FOR SCIENTIFIC REVIEW
CMGC
02/07/2022 - 02/08/2022

Notice of NIH Policy to All Applicants: Meeting rosters are provided for information purposes only. Applicant investigators and institutional officials must not communicate directly with study section members about an application before or after the review. Failure to observe this policy will create a serious breach of integrity in the peer review process, and may lead to actions outlined in NOT-OD-14-073 at https://grants.nih.gov/grants/guide/notice-files/NOT-OD-14-073.html, NOT-OD-15-106 at https://grants.nih.gov/grants/guide/notice-files/NOT-OD-15-106.html, and NOT-OD-18-115 at https://grants.nih.gov/grants/guide/notice-files/NOT-OD-18-115.html, including removal of the application from immediate review.

CHAIRPERSON(S)

BADGER, TERRY A, PHD, RN
PROFESSOR
COLLEGE OF NURSING
UNIVERSITY OF ARIZONA
TUCSON, AZ 85721

MEMBERS

BELANGER, EMMANUELLE, PHD *
ASSISTANT PROFESSOR
SCHOOL OF PUBLIC HEALTH
BROWN UNIVERSITY
PROVIDENCE, RI 02903

BROWN, ELLEN LESLIE, MSN, EDD *
ASSOCIATE PROFESSOR
NICOLE WERTHEIM COLLEGE OF NURSING AND HEALTH SCIENCES
FLORIDA INTERNATIONAL UNIVERSITY
MIAMI, FL 33199

DEAN, GRACE E, PHD
PROFESSOR
SCHOOL OF NURSING
STATE OF UNIVERSITY NEW YORK
UNIVERSITY AT BUFFALO
BUFFALO, NY 14214

EAKIN, MICHELLE NUTTALL, PHD
ASSOCIATE PROFESSOR
DEPARTMENT OF PULMONARY AND CRITICAL CARE MEDICINE
SCHOOL OF MEDICINE
JOHNS HOPKINS UNIVERSITY
BALTIMORE, MD 21224

FAN, VINCENT S, MPH, MD
ASSOCIATE PROFESSOR
DIVISION OF PULMONARY, CRITICAL CARE AND SLEEP MEDICINE
UNIVERSITY OF WASHINGTON
SEATTLE, WA 98195

HAMEL, LAUREN M., PHD *
ASSOCIATE PROFESSOR
COMMUNICATION AND BEHAVIORAL ONCOLOGY
POPULATION STUDIES/DISPARITIES RESEARCH PROGRAM
KARMANOS CANCER INSTITUTE, SCHOOL OF MEDICINE
WAYNE STATE UNIVERSITY
DETROIT, MI 48021

HAQUE, REINA, MPH, PHD
RESEARCH SCIENTIST III & SCIENTIFIC ADVISOR, CANCER REGISTRY
DEPARTMENT OF RESEARCH AND EVALUATION
KAISER PERMANENTE SOUTHERN CALIFORNIA
PASADENA, CA 91101

HARRELL, ERIN RENEE, PHD *
ASSISTANT PROFESSOR
DEPARTMENT OF PSYCHOLOGY
THE UNIVERSITY OF ALABAMA
TUSCALOOSA, AL 35487

JIMENEZ, MANUEL E, MD *
ASSISTANT PROFESSOR
DEPARTMENT OF PEDIATRICS
CHILD HEALTH INSTITUTE OF NEW JERSEY
RUTGERS UNIVERSITY
NEW BRUNSWICK, NJ 08901

KAIMAL, ANJALI, MAS, MD *
ASSOCIATE PROFESSOR
DEPARTMENTS OF OBSTETRICS AND GYNECOLOGY
AND POPULATION MEDICINE
MASSACHUSETTS GENERAL HOSPITAL
HARVARD MEDICAL SCHOOL
BOSTON, MA 02114

KELLEHER, SARAH, PHD *
ASSISTANT PROFESSOR
DEPARTMENT OF PSYCHIATRY AND BEHAVIORAL
SCIENCES
DUKE UNIVERSITY
DURHAM, NC 27705

KEMPER, ALEX R, MPH, MD
PROFESSOR
DEPARTMENT OF PEDIATRICS
NATIONWIDE CHILDREN'S HOSPITAL
THE OHIO STATE UNIVERSITY
COLUMBUS, OH 43205

KRANS, ELIZABETH E, MSC, MD
ASSOCIATE PROFESSOR
DEPARTMENT OF OBSTETRICS,
GYNECOLOGY AND REPRODUCTIVE SCIENCES
MAGEE-WOMEN'S HOSPITAL
UNIVERSITY OF PITTSBURGH MEDICAL CENTER
PITTSBURGH, PA 15213

LEVIN, JENNIFER BETH, PHD
ASSOCIATE PROFESSOR
DEPARTMENT OF PSYCHIATRY
SCHOOL OF MEDICINE
CASE WESTERN RESERVE UNIVERSITY
CLEVELAND, OH 44106

MAGWOOD, GAYENELL SMITH, RN, PHD
PROFESSOR
DEPARTMENT OF NURSING
COLLEGE OF NURSING
MEDICAL UNIVERSITY OF SOUTH CAROLINA
CHARLESTON, SC 29425

MILLER, LYNDSEY MEDORA, PHD *
ASSISTANT PROFESSOR
SCHOOL OF NURSING
OREGON HEALTH & SCIENCE UNIVERSITY
PORTLAND, OR 97239

NELSON, KERRIE P, PHD *
RESEARCH PROFESSOR
DEPARTMENT OF BIOSTATISTICS
SCHOOL OF PUBLIC HEALTH
BOSTON UNIVERSITY
BOSTON, MA 02118

NELSON, LONNIE A, PHD
ASSOCIATE PROFESSOR
COLLEGE OF NURSING
WASHINGTON STATE UNIVERSITY
SPOKANE, WA 99202

PERRY, TAMARA TAYLOR, MD
PROFESSOR OF PEDIATRICS
DEPARTMENT OF PEDIATRICS
UNIVERSITY OF ARKANSAS FOR MEDICAL SCIENCES
LITTLE ROCK, AR 72202

PIAMJARIYAKUL, UBOLRAT, PHD *
ASSOCIATE PROFESSOR
OFFICE OF RESEARCH AND SCHOLARLY ACTIVITIES
WEST VIRGINIA UNIVERSITY
MORGANTOWN, WV 26506-9600

PIATT, GRETCHEN A, MPH, PHD
ASSOCIATE PROFESSOR
DEPARTMENT OF LEARNING HEALTH SCIENCES
UNIVERSITY OF MICHIGAN
ANN ARBOR, MI 48109

POTTER, MICHAEL B, MD *
PROFESSOR
DEPARTMENT OF FAMILY AND COMMUNITY MEDICINE
SCHOOL OF MEDICINE
UNIVERSITY OF CALIFORNIA, SAN FRANCISCO
SAN FRANCISCO, CA 94121

RECKREY, JENNIFER M, MD *
ASSOCIATE PROFESSOR
DEPARTMENT OF GERIATRICS
AND PALLIATIVE MEDICINE
ICAHN SCHOOL OF MEDICINE AT MOUNT SINAI
NEW YORK, NY 10029

REID, CARY, MD, PHD *
PROFESSOR
DEPARTMENT OF MEDICINE
DIVISION OF GERIATRIC MEDICINE
WEILL CORNELL MEDICAL COLLEGE
NEW YORK, NY 10075

ROBERTO, KAREN A, PHD *
UNIVERSITY DISTINGUISHED PROFESSOR
CENTER FOR GERONTOLOGY
VIRGINIA POLYTECHNIC INSTITUTE & STATE UNIVERSITY
BLACKSBURG, VA 24061

ROLBIECKI, ABIGAIL JORDAN, MPH, MSW, PHD *
ASSISTANT PROFESSOR
SCHOOL OF MEDICINE
UNIVERSITY OF MISSOURI
COLUMBIA, MO 65212

SCHNALL, REBECCA, RN, MPH, PHD
MARY DICKEY LINDSAY ASSOCIATE PROFESSOR
HEALTH PROMOTION AND DISEASE PREVENTION
SCHOOL OF NURSING
COLUMBIA UNIVERSITY
NEW YORK, NY 10032

SONG, MI-KYUNG, FAAN, PHD
PROFESSOR
SCHOOL OF NURSING
EMORY UNIVERSITY
ATLANTA, GA 30322

STANFILL, ANSLEY, PHD *
ASSOCIATE PROFESSOR
ASSOCIATE DEAN OF RESEARCH
COLLEGE OF NURSING
THE UNIVERSITY OF TENNESSEE HEALTH SCIENCE CENTER
MEMPHIS, TN 38163

STEINHARDT, MARY A., EDD *
PROFESSOR
DEPARTMENT OF KINESIOLOGY AND HEALTH EDUCATION
THE UNIVERSITY OF TEXAS AT AUSTIN
AUSTIN, TX 78712

TISMINETZKY, MAYRA S., MPH, MD, PHD *
ASSOCIATE PROFESSOR
DEPARTMENT OF MEDICINE
UNIVERSITY OF MASSACHUSETTS MEDICAL SCHOOL
WORCESTER, MA 01605

TORKE, ALEXIA M, MD
PROFESSOR
CENTER OF AGING RESEARCH
INDIANA UNIVERSITY
INDIANAPOLIS, IN 46202

TUROK, DAVID, MPH, MD
ASSOCIATE PROFESSOR
DEPARTMENT OF OBSTETRICS AND GYNECOLOGY
UNIVERSITY OF UTAH
SALT LAKE CITY, UT 84132

VAUGHAN DICKSON, VICTORIA, MSN, PHD *
ASSOCIATE PROFESSOR
COLLEGE OF NURSING
NEW YORK UNIVERSITY
NEW YORK, NY 10003

WELLS, KRISTEN JENNIFER, MPH, PHD
PROFESSOR
DEPARTMENT OF PSYCHOLOGY
SAN DIEGO STATE UNIVERSITY
SAN DIEGO, CA 92120

WILLIAMS, KRISTINE N, PHD
E JEAN HILL PROFESSOR
SCHOOL OF NURSING
UNIVERSITY OF KANSAS MEDICAL CENTER
KANSAS CITY, KS 66160

WITTENBERG, ELAINE M, PHD *
ASSOCIATE PROFESSOR
DEPARTMENT OF COMMUNICATION STUDIES
CALIFORNIA STATE UNIVERSITY, LOS ANGELES
LOS ANGELES, CA 90032

SCIENTIFIC REVIEW OFFICER

FORDYCE, LAUREN, PHD
SCIENTIFIC REVIEW OFFICER
CENTER FOR SCIENTIFIC REVIEW
NATIONAL INSTITUTES OF HEALTH
BETHESDA, MD 20892

EXTRAMURAL SUPPORT ASSISTANT

ADEBONA, ADEBIMIBOLA O
LEAD EXTRAMURAL SUPPORT ASSISTANT
CENTER FOR SCIENTIFIC REVIEW
NATIONAL INSTITUTES OF HEALTH
BETHESDA, MD 20892

* Temporary Member. For grant applications, temporary members may participate in the entire meeting or may review only selected applications as needed.

Consultants are required to absent themselves from the room during the review of any application if their presence would constitute or appear to constitute a conflict of interest.
